# Supplementary material for: Predicting visual function by interpreting a neuronal wiring diagram
Source: Nature. 2024 Oct 2;634(8032):113–23. doi: 10.1038/s41586-024-07953-5 (PMC11446822; doi:10.1038/s41586-024-07953-5)
Supplement: Supplementary file 6 — CRF and ERF predictions for individual TmY4 and TmY9 cells. Analogous to Supplementary Data 3, but for TmY target types. Shown are the top four monosynaptic pathways, the strongest pathway passing through each of the top ten intermediary types (ranking from Extended Data Fig. 7), and the trisynaptic pathway Tm1–TmY–Dm3–TmY (see the section entitled Prediction of spatial normalization). [file 41586_2024_7953_MOESM6_ESM.zip › DataS4/TmY9qΓèÑ/720575940612532709.pdf]

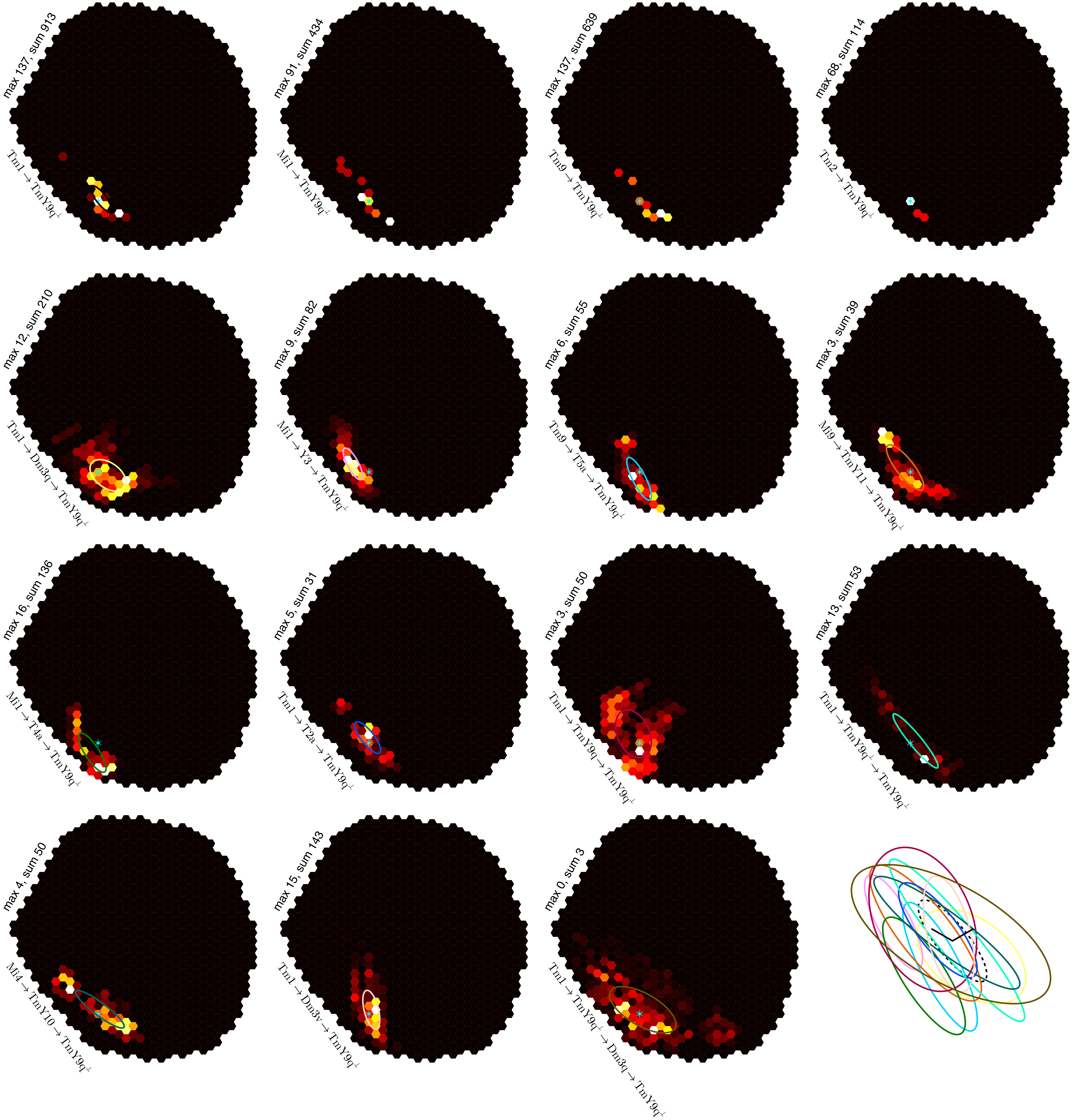

max 137, sum 913
Tm1 → TmY9q⊥
max 91, sum 434
Mi1 → TmY9q⊥
max 137, sum 639
Tm9 → TmY9q⊥
max 68, sum 114
Tm2 → TmY9q⊥
max 12, sum 210
Tm1 → Dm3q → TmY9q⊥
max 9, sum 82
Mi1 → Y3 → TmY9q⊥
max 6, sum 55
Tm9 → T5a → TmY9q⊥
max 3, sum 39
Mi9 → TmY11 → TmY9q⊥
max 16, sum 136
Mi1 → T4a → TmY9q⊥
max 5, sum 31
Tm1 → T2a → TmY9q⊥
max 3, sum 50
Tm1 → TmY9q → TmY9q⊥
max 13, sum 53
Tm1 → TmY9q⊥ → TmY9q⊥
max 4, sum 50
Mi4 → TmY10 → TmY9q⊥
max 15, sum 143
Tm1 → Dm3v → TmY9q⊥
max 0, sum 3
Tm1 → TmY9q⊥ → Dm3q → TmY9q⊥
